# Thyroid Cancer-Associated Mitochondrial DNA Mutation G3842A Promotes Tumorigenicity via ROS-Mediated ERK1/2 Activation

**DOI:** 10.1155/2022/9982449

**Published:** 2022-03-15

**Authors:** Sixi Chen, Xinzhu Bao, Hongzhi Chen, Manli Jia, Wen Li, Luyi Zhang, Runxiao Fan, Hezhi Fang, Liqin Jin

**Affiliations:** ^1^Clinical Laboratory Center, The Second Affiliated Hospital and Yuying Children's Hospital of Wenzhou Medical University, Wenzhou, Zhejiang 325027, China; ^2^Department of Clinical Laboratory, People's Hospital of Deyang City, Deyang, China; ^3^National Clinical Research Center for Metabolic Diseases, Key Laboratory of Diabetes Immunology, Ministry of Education and Department of Metabolism and Endocrinology, The Second Xiangya Hospital of Central South University, 139 Middle Renmin Road, Changsha, 410011 Hunan, China; ^4^Key Laboratory of Laboratory Medicine, Ministry of Education, Zhejiang Provincial Key Laboratory of Medical Genetics, College of Laboratory Medicine and Life sciences, Wenzhou Medical University, Wenzhou, Zhejiang 325035, China; ^5^Zhengjiang Provincial People's Hospital, Affiliated People's Hospital of Hangzhou Medical College, Hangzhou, Zhejiang, China

## Abstract

Mitochondrial DNA (mtDNA) mutations have been identified in various human cancers, including thyroid cancer. However, the relationship between mtDNA and thyroid cancer remains unclear. Previous studies by others and us strongly suggested that mtDNA mutations in complex I may participate in thyroid cancer processes according to sequencing results of thyroid cancer tissue, although the associated pathogenic processes remain unknown. Here, to investigate whether mtDNA mutations contribute to thyroid cancer, we reanalyzed our sequencing results and characterized thyroid cancer-associated mutations in the mitochondrial complex. The results identified the highest mutation frequencies in nicotinamide adenine dinucleotide hydride (NADH) dehydrogenase subunit 4 gene (*ND4*) and cytochrome *c* oxidase subunit 1 gene (*COI*), which also harbored the highest rates of G > A substitutions, with most of the mutations resulting in changes in the polarity of amino acids. We then established cybrids containing the G3842A mutation identified in papillary thyroid carcinoma, which revealed it as a mutation in NADH dehydrogenase subunit 1 gene (*ND1*) and is previously reported in follicular thyroid carcinoma, thereby suggesting a possibly pathogenic role in thyroid carcinoma. Additionally, we found that the G3842A mutation accelerates tumorigenicity and decreases the abundance and activity of mitochondrial complex I, the oxygen consumption rate, and adenosine triphosphate levels. By contrast, the levels of reactive oxygen species (ROS) were increased to activate extracellular signal-regulated kinase (ERK1/2) signaling, which contributed to tumorigenicity. These findings suggest for the first time that mtDNA mutations help drive tumor development and that G3842A may represent a new risk factor for thyroid cancer. Furthermore, our findings indicate that drugs targeting ROS and ERK1/2 may serve as a viable therapeutic strategy for thyroid cancer.

## 1. Introduction

Mammalian mitochondria contain an independent genome referred to as mitochondrial DNA (mtDNA) that comprises 16,569 nucleotide pairs encoding 13 essential polypeptides of the respiratory chain complex, as well as two rRNAs and 22 tRNAs necessary for gene expression [1]. Compared with nuclear DNA, mtDNA is more susceptible to oxidative damage and has a higher mutation rate because of its proximity to reactive oxygen species (ROS) produced by the respiratory chain, lack of histone proteins, and limited DNA repair activity [2] [3]. Somatic mutations in mtDNA are frequently detected in cancerous tissues, including liver [4], breast [5], colorectal [6], prostate [7], and thyroid [8] cancers, although few functional studies of these mutations have been described. Thus, it remains a matter of debate as to whether mtDNA mutations represent oncogenic events or byproducts of tumorigenesis.

In recent years, the incidence of thyroid cancer has increased rapidly worldwide [9]; however, the mechanisms underlying thyroid cancer development are not completely elucidated. The most well-known genetic alteration is the BRAF V600E transversion, which is the most common BRAF mutation in thyroid cancer and is now used clinically to assess the risk of thyroid cancer metastasis and prognosis [10]. BRAF is a cytoplasmic serine-threonine kinase, a member of the RAS/RAF/MEK/ERK mitogen-activated protein kinase (MAPK) cell signaling pathway [11]. The BRAF V600E mutation leads to continuous activation of the MAPK pathway to promote oncogenic transformation [12]. Additionally, it is generally believed that the incidence of thyroid cancer is related to inherited genetic syndromes, radiation exposure, and hormone and iodine deficiencies [13].

During the previous decade, the relationship between thyroid cancer and mtDNA attracted increasing attention. Previous studies sequenced samples of thyroid cancer patients and identified mtDNA mutations mostly in genes related to mitochondrial complex I [14, 15]. Notably, in 2018, Ganly et al. [16] and Gopal et al. [17] performed genomic analyses and found that mtDNA mutations enriched in genes of complex I may be drivers of Hürthle cell cancer. Consistent with their conclusions, we previously found a statistically significant difference in the mtDNA mutation rate in complex I between thyroid carcinoma and benign thyroid tumors [18]. However, whether and how mtDNA mutations contribute to the development of thyroid cancer remain largely unknown.

G3842A is a nonsense mutation in nicotinamide adenine dinucleotide hydride (NADH) dehydrogenase (ND) subunit 1 gene (*ND1*), which encodes a part of complex I. We previously identified this mutation in papillary thyroid carcinoma [18], and it has also been identified in follicular thyroid carcinoma (FTC) [15] and liver cancer [19], suggesting the pathogenicity of G3842A. Therefore, in the present study, we reanalyzed our sequencing results in order to evaluate a possible link between mtDNA and tumorigenicity in thyroid cancer and established G3842A mutation cybrids to investigate the influence of G3842A on tumor development.

## 2. Materials and Methods

### 2.1. Mutation Reanalysis

Somatic mitochondrial mutations were identified in a previous study [18]. Briefly, we collected pathological specimens and blood from patients with thyroid carcinoma, follicular thyroid adenoma, or nodular goiter in Wenzhou, a city with a high incidence of thyroid cancer in Zhejiang province, China. Carcinomatous and paracancerous tissues in pathological sections were distinguished under a microscope. We extracted and sequenced DNA from carcinomatous and paracancerous tissues and blood, and DNA sequencing results were analyzed using CodonCode Aligner (v.4.0.4; CodonCode Corp., Centerville, MA, USA), with the revised Cambridge reference sequence used as the reference sequence. We defined somatic mtDNA mutation sites as sequence changes occurring in the diseased tissues but not the blood. In the present study, Supplementary table S1 was obtained by filtering out the somatic mtDNA mutation sites considered as polymorphic sites in the Human Mitochondrial Genome Database to determine the tumorigenicity of mtDNA mutations.

### 2.2. Cell Lines and Culture Conditions

Cybrids were obtained by transforming platelets of patients containing the G3842A mutation into mtDNA-less p0 human osteosarcoma 143b cells, as described previously [20]. Because of the heterogeneity of the G3842A mutation in cybrids, we screened 143b cybrid cells only containing 3842G as the wild-type (WT) group and 143b cybrid cells containing 3842A as the MUT group, thus ensuring that the WT and G3842A mutation groups had the same genetic background, except for the G3842A mutation. Cells were cultured in high-glucose Dulbecco's modified Eagle medium (DMEM) (Thermo Fisher Scientific, Waltham, MA, USA) containing 12% cosmic calf serum (Sigma, Aldrich, St. Louis, MO, USA) at 37°C.

### 2.3. Cell Proliferation Assays

Cells were seeded in six-well plates at a density of 10 × 10^4^ cells/well. Cell culture medium was replaced every 2 days, and the number of cells was counted using NovoCyte flow cytometry (Agilent Technologies, Santa Clara, CA, USA) every 24 h.

### 2.4. Colony Formation Assays

Cells were seeded in six-well plates at a density of 1 × 10^3^ cells/well, with the culture medium being replaced every 3 days. After 14 days, the clones were fixed with 4% paraformaldehyde (Shanghai Lingfeng Chemical Reagent, Shanghai, China) and stained with crystal violet (Beyotime, Beijing, China). Colonies were photographed and quantified using ImageJ software (NIH, Bethesda, MD, USA).

### 2.5. Wound Healing, Cell migration, and Cell Invasion Assays

Wound healing, cell migration, and cell invasion assays were performed as previously described [21].

### 2.6. Polymerase Chain Reaction-Restriction Fragment Length Polymorphism (PCR-RFLP)

DNA was extracted from cells as previously described [22]. The PCR primer pairs were F-(5′-TACTTCACAAAGCGCCTTCC-3′) and R-(5′-ATGAAGAATAGGGCGAAGGG-3′). PCR products were digested with 10 U of BspHI (New England Biolabs, Ipswich, MA, USA) to form two fragments (693 bp and 139 bp for WT mtDNA) or one fragment (832 bp for mutant mtDNA).

### 2.7. Immunoblotting and Antibodies

Total proteins and mitochondrial membrane proteins were isolated and separated as previously described [21]. Proteins were probed using anti-ERK1/2 (Cell Signaling Technology, Danvers, MA, USA), anti-phosphorylated (p)-ERK (Thr202/Tyr204; Cell Signaling Technology), anti-p38 (Cell Signaling Technology), anti-phospho-p38 (Thr389; Cell Signaling Technology), anti-c-Jun N-terminal protein kinase (JNK; Cell Signaling Technology), anti-phospho-JNK (Cell Signaling Technology), anti-GRIM19 (Abcam, Cambridge, UK), anti-SDHA (Abcam), anti-UQCRC2 (Abcam), anti-MT-COI (Abcam), and anti-ATP-5A (Abcam) antibodies. Probed proteins were incubated with anti-rabbit (Cell Signaling Technology) or anti-mouse IgG (Cell Signaling Technology) secondary antibodies. Blot signals were detected using Super Signal West Pico Chemiluminescent substrate (Thermo Fisher Scientific).

### 2.8. ATP Measurements

The ATP content in WT and G3842A mutant cells was measured using an ATP measurement kit (Thermo Fisher Scientific) according to the manufacturers' instructions.

### 2.9. Calcium Ion (Ca^2+^) Measurements

WT and G3842A mutant cells were incubated in 4 *μ*M Rhod 2-AM (Abcam) working solution. Fluorescence was detected using a microplate reader (Molecular Devices, San Jose, CA, USA), with excitation and emission wavelengths of 557 nm and 581 nm, respectively.

### 2.10. ROS Measurements

ROS levels were measured using flow cytometry according to a previously published protocol [23]. ROS was measured by staining cells with 5 *μ*M MitoSOX (Thermo Fisher Scientific) or 20 *μ*M 6-chloromethyl-2′,7′-dichlorodihydrofluorescein diacetate acetyl ester (carboxy-H2DCFDA; Thermo Fisher Scientific). Data were normalized according to cell numbers, and results are presented as the mean fluorescence intensity relative to WT cells.

### 2.11. Transcriptome Sequencing and Analysis

Total RNA was extracted from cells with and without the G3842A mutation, and transcriptome sequencing was performed by Novogene (Nanjing, China), as previously described [24]. Gene expression was analyzed using DESeq2 R (https://bioconductor.org/packages/release/bioc/html/DESeq2.html), and an adjusted *P* < 0.05 was used to select differentially expressed genes (DEGs).

### 2.12. Oxygen Consumption Rate (OCR) Measurements

OCR in WT and G3842A mutant cells were determined using an XF24 extracellular flux analyzer (Seahorse Bioscience, Santa Clara, CA, USA), as previously described [25]. After calibration, the basic respiration, ATP production (using 1 *μ*M oligomycin; BBI Life Science, Shanghai, China), and maximum respiratory capacity (using 0.5 *μ*M carbonyl cyanide-p-trifluoromethoxy phenylhydrazone; Sigma-Aldrich) were measured.

### 2.13. Complex I-Dependent Respiration Measurements

Complex I-dependent respiration was measured using a Clark-type oxygen electrode (Hansatech, Norfolk, UK). After recording the basal respiration, glutamine and malate substrates were added to measure the oxygen consumption of complex I-dependent respiration, with rotenone added to stop complex I activity. The results were normalized against the number of cells added to the Clark-type oxygen electrode for each group.

### 2.14. Xenograft Experiments

Animal experiments were performed in accordance with the guide for care and use of laboratory animals outlined by the Animal Ethics Committee of Wenzhou Medical University. Nude mice (5 weeks old; Beijing Vital River Laboratory Animal Technology, Shanghai, China) were randomly divided into four groups: WT, WT + N-acetyl-L-cysteine (WT + NAC), mutant (MUT), and MUT + NAC (*n* = 6/group). The WT and WT + NAC groups received subcutaneous injection into the right side of the neck with 2 × 10^6^ 143b cells without G3842A mutation, whereas the MUT and MUT + NAC groups received similar injections with 2 × 10^6^ 143b cells with the G3842A mutation. After 1 week, the WT + NAC and MUT + NAC groups were intraperitoneally injected with 100 mg/kg NAC twice a week, whereas the WT and MUT groups were intraperitoneally injected with NaCl. Each mouse developed only one visible tumor at the injection site. Tumor size was monitored weekly with a caliper, and tumor volume (*V*) was estimated using the formula: *V* = (length × width^2^) × 0.5236. Mice were weighed once a week, and tumor volumes were recorded on days 0, 7, 14, 21, 28, and 35 after treatment. All mice were sacrificed on day 35.

### 2.15. Reagents

NAC and U0126 were purchased from Sigma-Aldrich and Selleck Chemicals (Houston, TX, USA), respectively.

### 2.16. Statistical Analysis

All experiments were independently repeated at least three times. Data are presented as the mean ± standard error of the mean. Statistical significance was evaluated using one-way analysis of variance or an independent two-tailed Student's *t*-test using SPSS (v.21.0; IBM, Armonk, NY, USA). A *P* < 0.05 was considered significant.

## 3. Results

### 3.1. Genomic Characterization of Thyroid Cancer Mutations in the Mitochondrial Complex

**Figure 1:**
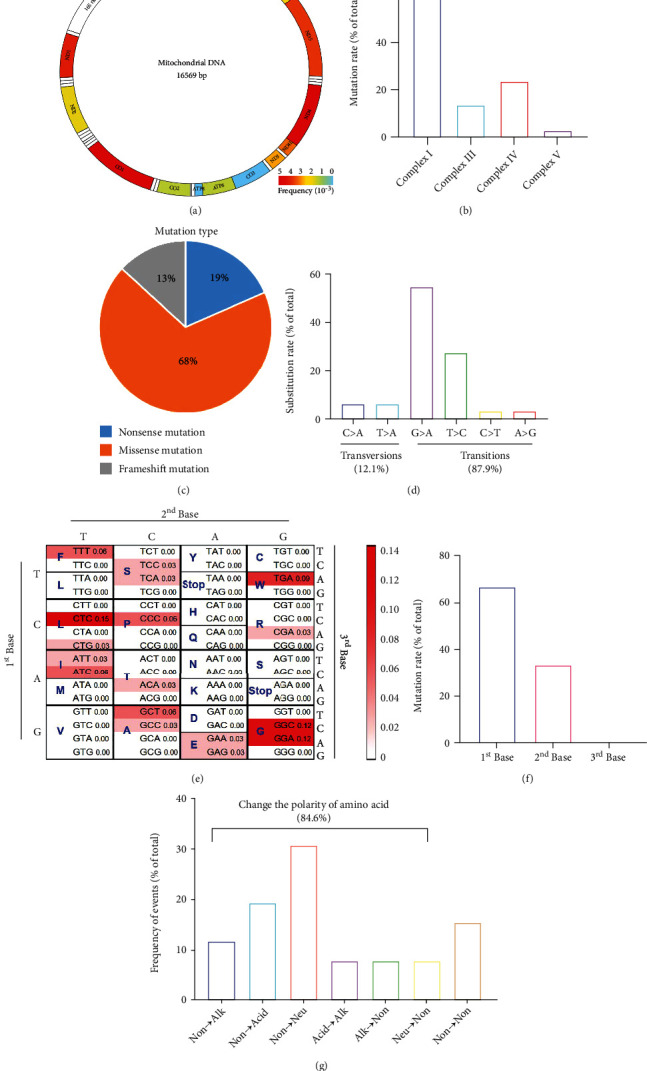
Genomic characterization of mitochondrial (mt) DNA mutations identified in patients with thyroid carcinoma. (a) Distribution and frequency of mtDNA-mutated genes encoding mitochondrial complex enzymes. The mutation frequency is shown with different colors. (b) Frequency of mtDNA mutations in mitochondrial complexes I, II, III, and IV. (c) Proportion of mutation types in identified mtDNA mutations. (d) Rate of different base substitutions in nonsense and missense mutations. The proportions of base transitions and base transversions are also indicated. (e) Frequency of mtDNA mutations in different triplet codons. The mutation frequency is shown with different colors. (f) Mutation rates of the first, second, and third bases. (g) Frequencies of different types of changes in the polarity of amino acids. Non: nonpolar amino acid; Alk: alkaline amino acid; Acid: acidic amino acid; Neu: neutral amino acid.

mtDNA mutations, especially those enriched in complex I genes, are considered potential genetic drivers in thyroid cancer [16, 17]. To obtain additional information about the characteristics of thyroid cancer mutations in mitochondrial complexes, we filtered and reanalyzed the mtDNA mutations reported in our previous study (S1). We found that the genes with the highest frequency of mtDNA mutations were *ND4* and *cytochrome c oxidase subunit 1* (*CO1*), followed by *ND1* and *cytochrome b* (*CYB*) (Figure 1(a)), with the majority of mtDNA mutations occurring in complex I (Figure 1(b)). Among the mutation types identified, missense mutations accounted for 68%, nonsense mutations 19%, and frameshift mutations 13% (Figure 1(c)). The base substitutions were most likely to occur in G > A, and the base transitions were more frequent than base transversions (Figure 1(d)). We found that mtDNA mutations occurred more frequently in the triplet codons GGA, GGC, and CTC (Figure 1(e)), with the first base of the codon having a higher probability of mutation (Figure 1(f)). Furthermore, we found that 84.6% of the mutations changed the polarity of the amino acid (Figure 1(g)).

### 3.2. G3842A Mutation Causes a Disruptive Change in Protein Structure

**Figure 2:**
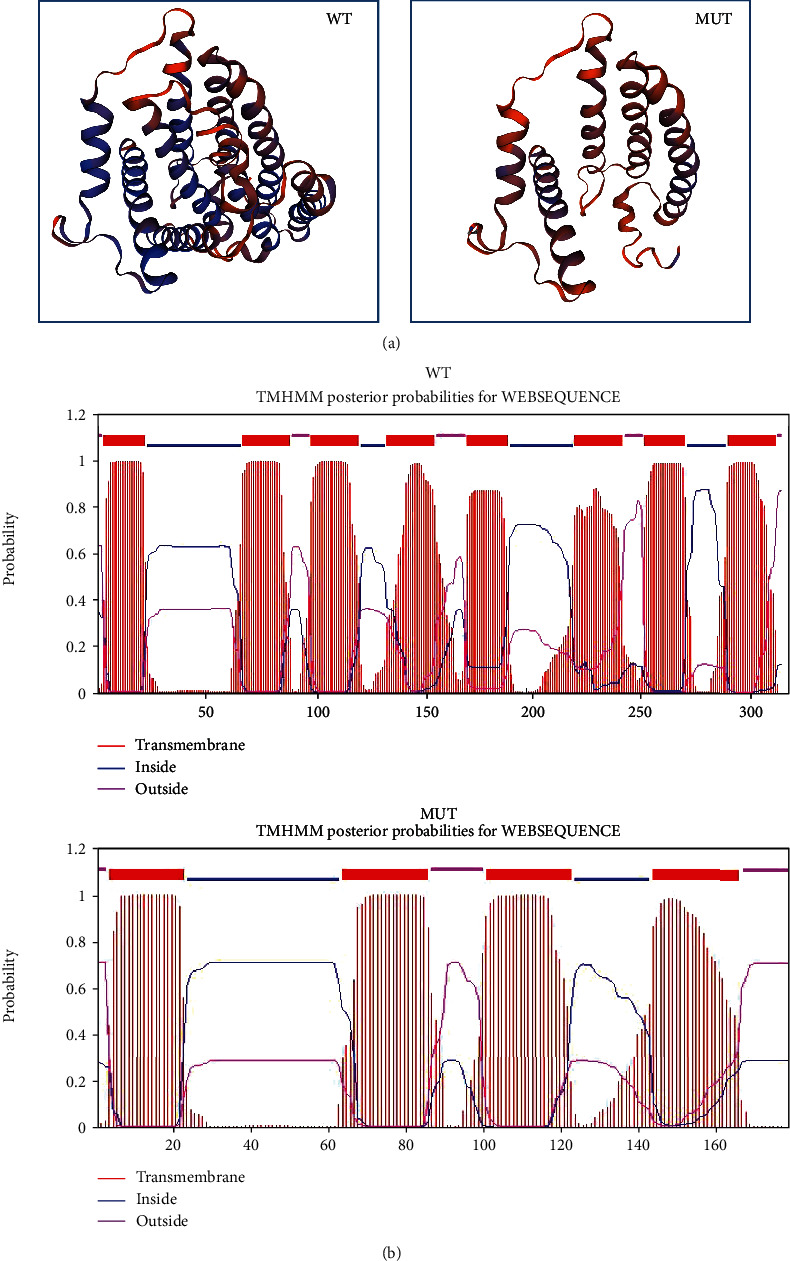
Effects of the G3842A mutation on the protein structure. (a) Structures of proteins encoded by *NADH dehydrogenase subunit 1* (*ND1*) and *ND1* harboring the G3842A mutation were predicted using the SWISS-MODEL. (b) Transmembrane structures of proteins encoded by *ND1* and *ND1* harboring the G3842A mutation were obtained from TMHMM. NADH: nicotinamide adenine dinucleotide hydride.

Among the mutations identified in complex I genes, G3842A has also been identified in FTC and liver cancer, suggesting the pathogenicity of this mutation. We then used the SWISS-MODEL (https://swissmodel.expasy.org/interactive) to predict how the G3842A mutation affects the protein structure.  Figure 2(a) shows that G3842A creates a premature stop codon in *ND1*, which generates a truncated ND1 polypeptide. Because ND is a transmembrane protein, we determined the transmembrane structure using TMHMM (http://www.cbs.dtu.dk/services/TMHMM/). As shown in Figure 2(b), the G3842A mutation reduced the number of transmembrane domains from eight to four.

### 3.3. G3842A Mutation Promotes Tumorigenicity In Vitro

**Figure 3:**
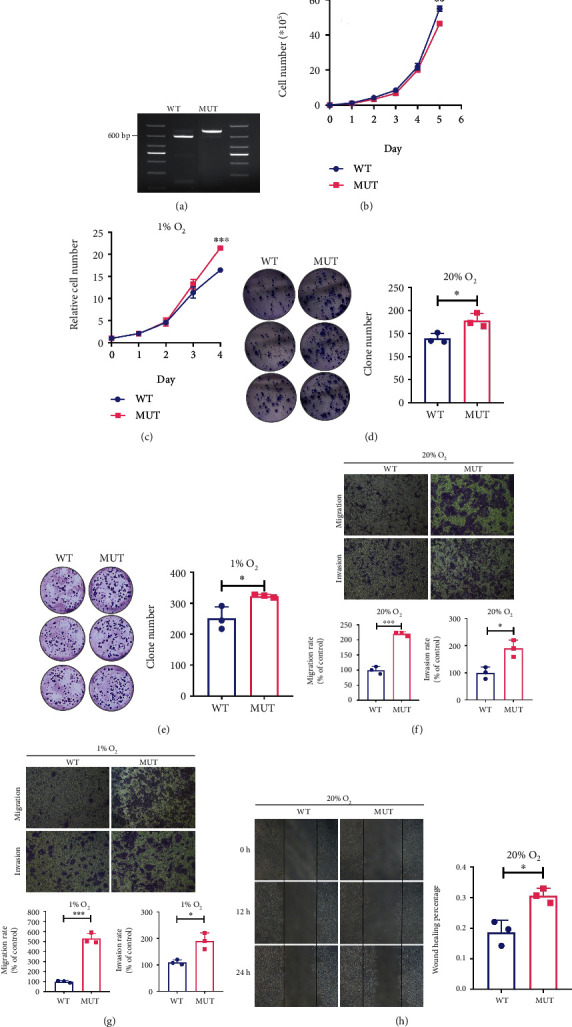
Effects of the G3842A mutation on tumorigenicity *in vitro*. (a) PCR-RFLP assays confirmed the established 143b cell line with G3842A mutation (MUT). After enzymatic digestion, DNA from 143b cells without the G3842A mutation (WT) yielded fragments of 693 bp and 139 bp. 143b cells with and without G3842A mutation were cultured *in vitro* under (b) 20% and (c) 1% O_2_ conditions, and the cell number was counted daily. Colony formation assays showing increased proliferation of MUT cells as compared with WT cells under both (d) 20% and (e) 1% O_2_ conditions. Transwell assays were performed to evaluate the migration and invasion abilities of MUT and WT cells under (f) 20% and (g) 1% O_2_ conditions. (h) Wound healing assays further validated the increased migration ability of MUT cells. The wound healing capacity was calculated as follows: [(exposed area at 0 h − exposed area at 24 h)/exposed area at 0 h]. Data are presented as mean ± SEM of at least three replicates. ^∗^*P* < 0.05, ^∗∗^*P* < 0.01, and ^∗∗∗^*P* < 0.001. PCR-RFLP: polymerase chain reaction-restriction fragment length polymorphism; SEM: standard error of the mean.

We established a 143b cell line model with the G3842A mutation and confirmed its establishment with PCR-RFLP assays (Figure 3(a)). We then performed experiments under normoxic and hypoxic conditions to detect the effects of G3842A on tumorigenicity *in vitro*, finding that under 20% O_2_ condition, the cell number of the 143b cybrids with the G3842A mutation (MUT) was similar to that of 143b cybrids without the G3842A mutation (WT) after a 5-day cultivation (Figure 3(b)), although under 1% O_2_ condition, the cell number of the MUT was higher than that of the WT (Figure 3(c)). Moreover, compared with WT, the G3842A mutation accelerated clonal formation regardless of the O_2_ condition (Figures 3(d) and 3(e)). Additionally, the G3842A mutation significantly increased the migration and invasion rates of 143b cells under both O_2_ conditions (Figures 3(f) and 3(g)), with wound healing assays showing that the MUT demonstrated higher cell migration relative to the WT (Figure 3(h)).

### 3.4. G3842A Mutation Impairs Mitochondrial Oxidative Phosphorylation (OXPHOS) Function

**Figure 4:**
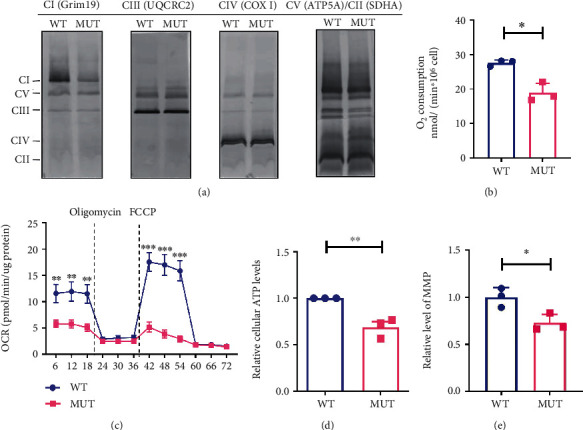
Effects of the G3842A mutation on mitochondrial function. (a) Measurements of the content of mitochondrial complexes I, II, III, IV, and V in WT and MUT using BN-PAGE and immunoblotting. (b) Measurements of complex I-dependent oxygen respiration in WT and MUT. (c) The OCR of WT and MUT was determined using an XF24 extracellular flux analyzer and initially under basal conditions, followed by the addition of oligomycin (100 *μ*g/mL) and FCCP (50 *μ*g/mL). Measurements of (d) ATP levels and (e) MMP levels in WT and MUT cells. The OCR and ATP generation were normalized against protein concentrations. Data are presented as mean ± SEM of at least three replicates. ^∗^*P* < 0.05, ^∗∗^*P* < 0.01, and ^∗∗∗^*P* < 0.001. BN-PAGE: blue native polyacrylamide gel electrophoresis; OCR: oxygen consumption rate; ATP: adenosine triphosphate; MMP: mitochondrial membrane protein; FCCP: carbonyl cyanide-p-trifluoromethoxy phenyl hydrazine.

We then measured the OXPHOS function in WT and MUT and found that the G3842A mutation decreased complex I levels, whereas complexes II, III, IV, and V were unaffected (Figure 4(a)). Additionally, the oxygen consumption of complex I was decreased in MUT as compared with WT (Figure 4(b)). We then tested mitochondrial respiration and found that basal mitochondrial respiration, ATP production, and uncoupled mitochondrial respiration were all significantly decreased in MUT according to OCR analysis (Figure 4(c)). Consistent with the impaired OXPHOS function, ATP levels (Figure 4(d)) and mitochondrial membrane potential (MMP) in MUT were lower than those in WT (Figure 4(e)).

### 3.5. G3842A Mutation Induces the Generation of Messenger Molecules

**Figure 5:**
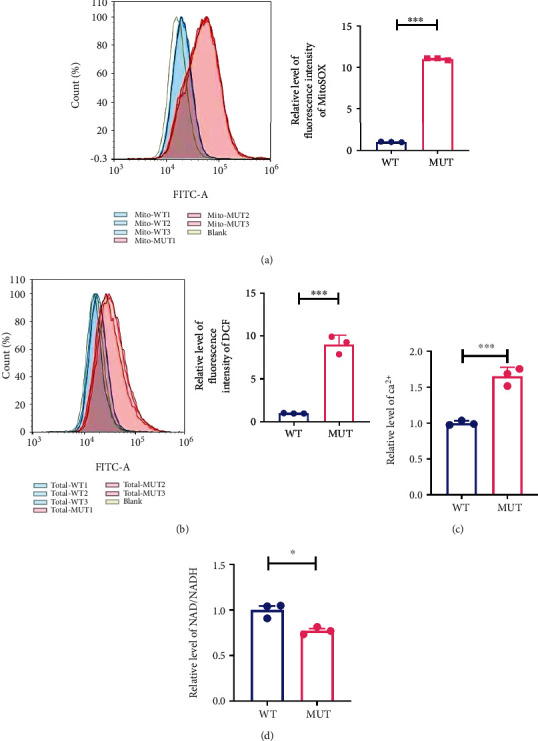
Effects of the G3842A mutation on messenger molecules. ROS levels are indicated by the relative levels of fluorescence intensity of MitoSOX (a) and DCF (b) in WT and MUT cells. Ca^2+^ (c) and the NAD/NADH ratio (d) were determined in WT and MUT cells. Signals for WT, MUT, and blank are shown in blue, red, and white, respectively. The horizontal coordinate corresponding to the signal peak represents the fluorescence intensity. We defined mean fluorescence intensity of WT as “1”: [(WT1 − blank) + (WT2 − blank) + (WT3 − blank)]/3. Relative fluorescence intensity was obtained by comparing the fluorescence intensity of MUT (after subtracting the fluorescence intensity of the blank) with the mean fluorescence intensity of WT, for example, (MUT1 − blank)/the mean fluorescence intensity of WT. Data are presented as mean ± SEM of at least three replicates. ^∗^*P* < 0.05, ^∗∗^*P* < 0.01, and ^∗∗∗^*P* < 0.001. ROS: reactive oxygen species; NADH: nicotinamide adenine dinucleotide hydride.

Mitochondrial dysfunction results in altered mitochondrial signaling; therefore, we determined changes in the levels of ROS, Ca^2+^, and the NAD/NADH ratio. Specifically, we used the fluorescent dye MitoSOX to detect mitochondrial ROS (especially superoxide) and carboxy-H2DCFDA was used to determine overall intracellular ROS levels, including hydrogen peroxide, hydroxyl radicals, and peroxynitrite. According to the fluorescence intensity, we found elevated ROS levels in the MUT relative to the WT (Figure 5(a)). Additionally, Ca^2+^ levels in the MUT were higher than those in WT (Figure 5(c)) and the NAD/NADH ratio decreased in MUT (Figure 5(d)).

### 3.6. G3842A Mutation Significantly Activates ERK1/2 Signaling

**Figure 6:**
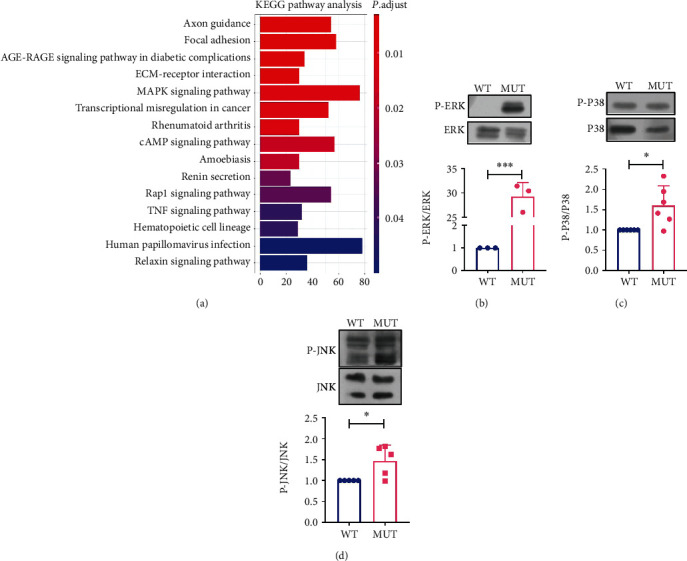
The G3842A mutation significantly activates ERK1/2 signaling. (a) Analysis of DEGs using the KEGG pathway database after transcriptome sequencing of WT and MUT cells. Phosphorylation levels of members of the MAPK pathway, including (b) ERK1/2, (c) p38, and (d) JNK in WT cells and MUT cells, were examined using Western blotting. All data are presented as mean ± SEM of at least three replicates. ^∗^*P* < 0.05, ^∗∗^*P* < 0.01, and ^∗∗∗^*P* < 0.001. DEGs: differentially expressed genes; KEGG: Kyoto Encyclopedia of Genes and Genomes; MAPK: mitogen-activated protein kinase; ERK: extracellular signal-regulated kinase; JNK: c-Jun N-terminal kinase.

Transcriptome sequencing of MUT and WT identified 3,900 DEGs that were subsequently analyzed using the Kyoto Encyclopedia of Genes and Genomes (KEGG) pathway database (Figure 6(a)). Pathway enrichment analysis revealed several signaling pathways associated with the G3842A mutation. Considering that MAPK is a major pathway associated with thyroid cancer [26], we evaluated changes in phosphorylation levels of P38, JNK, and ERK1/2 using Western blot. The results revealed higher levels of p-P38, p-JNK, and p-Erk1/2 in MUT relative to WT, with p-ERK1/2 showing the greatest increases (Figures 6(b)–6(d)).

### 3.7. G3842A Mutation Promotes Tumorigenicity via Increased ROS Generation and Subsequent ERK1/2 Phosphorylation

**Figure 7:**
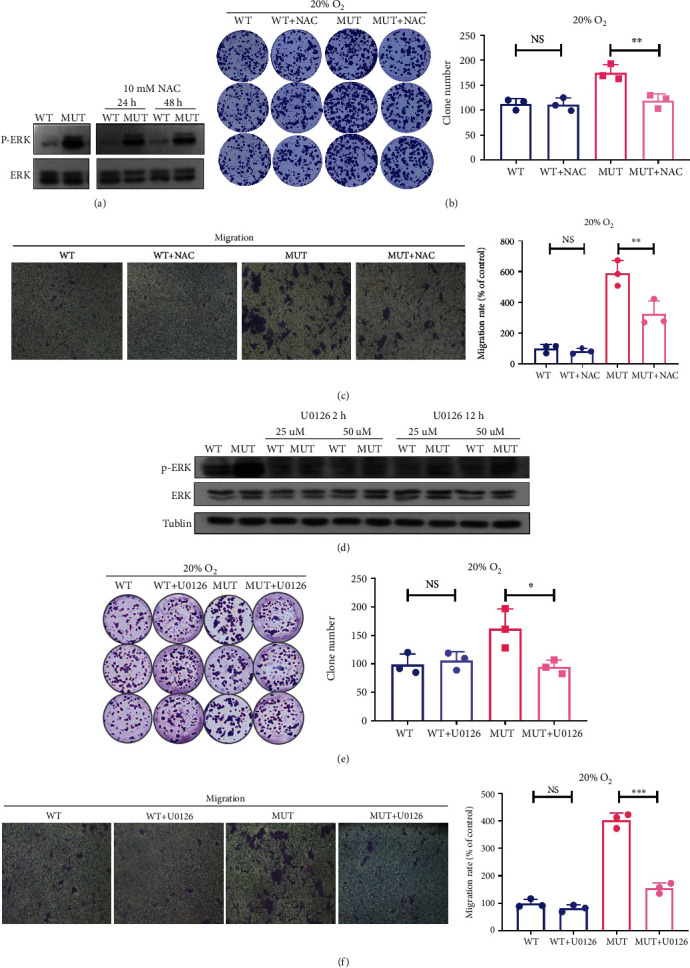
The G3842A mutation promotes tumorigenicity via increased ROS generation and subsequent ERK1/2 phosphorylation. (a) Measurements of p-ERK1/2 levels in WT and MUT treated with NAC (10 mM for 24 or 48 h) or without NAC using western blot. (b) Colony formation assays of WT and MUT treated with or without NAC. (c) The migration abilities of WT and MUT treated with or without NAC were test using Transwell assays. (d) p-ERK1/2 levels in WT and MUT treated with U0126 (25 or 50 *μ*m for 2 or 12 h) or without U0126 were evaluated by Western blot. (e) Colony formation assays and (f) Transwell assays of WT and MUT treated with or without U0126. Data are presented as mean ± SEM of at least three replicates. ^∗^*P* < 0.05, ^∗∗^*P* < 0.01, and ^∗∗∗^*P* < 0.001. NAC: N-acetyl-L-cysteine.

Previous studies reported marked increases in ERK signaling in BRAF-mutant thyroid cancer and found it to be associated with cancer development and treatment [27]. Therefore, we choose the ERK signaling pathway for further study. The ERK pathway is activated in response to increased ROS levels and participates in various cellular processes, including proliferation, migration, and apoptosis, that can also positively affect cancer development [28] [29]. Thus, we investigated whether the ROS-ERK1/2 pathway is also involved in the tumorigenicity of G3842A mutant cell. NAC has an antioxidant effect on all types of ROS. To investigate whether enhanced ROS production contributes to ERK1/2 phosphorylation, we treated WT and MUT cells with 10 mM NAC to scavenge ROS and then detected the phosphorylation level of ERK. The results showed that p-ERK1/2 levels decreased in MUT following 10 mM NAC treatment (Figure 7(a)). Additionally, we found that MUT treated with 10 mM NAC formed fewer clones relative to untreated MUT, whereas no difference in clone number was observed between NAC-treated and untreated WT (Figure 7(b)). Moreover, the migration rate of NAC-treated MUT was lower than that of untreated MUT (Figure 7(c)). These results indicated that activation of ERK1/2 signaling and the resulting increase in cell proliferation and migration triggered by the G3842A mutation might be driven at least partially through elevations in ROS levels.

Then, to determine the role of ERK1/2 signaling in G3842A mutation-related effects, we used U0126, a MEK1/2 inhibitor, which blocks ERK1/2 phosphorylation [30]. We treated cells with 25 *μ*M and 50 *μ*M U0126 for 2 h and 12 h and found that treatment of MUT with 25 *μ*M for 2 h is enough to attenuate the observed increases in ERK1/2 phosphorylation (Figure 7(d)). We then treated WT and MUT with 25 *μ*M U0126 and found that increased colony formation (Figure 7(e)) and migration (Figure 7(f)) were absent from G3842A mutant cells, whereas no differences in clone number and the migration rate were observed between U0126-treated and untreated WT. These findings indicated that G3842A promoted cell tumorigenicity that is dependent at least in part on ERK1/2 signaling.

Together, in MUT cells, elevated ROS levels facilitated ERK1/2 phosphorylation, which contributed to increased colony formation and migration.

### 3.8. G3842A Mutation Promotes ROS-Dependent Tumor Growth In Vivo

**Figure 8:**
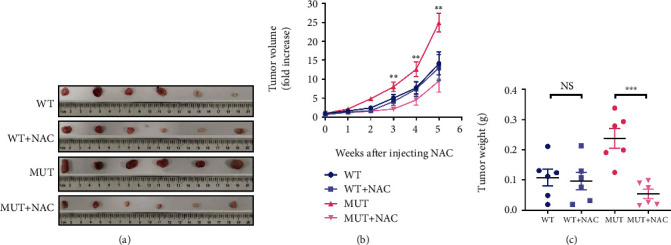
The G3842A mutation promotes tumor growth *in vivo* in a ROS-dependent manner. (a) Tumor xenograft experiments were performed by injecting nude mice with MUT cells or MUT + NAC cells or WT cells or WT + NAC cells. Each mouse developed only one visible tumor at the injection site. (b) Tumor volume was monitored weekly, and (c) tumor weight was calculated on 35 days after injection for each group (*n* = 6/group). The first NAC injection (shown as the 0 week after injecting NAC in the graph) was performed 1 week after subcutaneous injection of the cells, with the tumor volume measured before NAC injection. We defined the mean tumor volume of six mice on day 0 of NAC injection as “1.” The tumor volume of each mouse on weeks 1 through 5 after NAC injection was compared with the mean tumor volume of six mice (in each group) on day 0 of NAC injection in order to present increasing trends in tumor volume for each group. All data are presented as mean ± SEM. ^∗^*P* < 0.05, ^∗∗^*P* < 0.01, and ^∗∗∗^*P* < 0.001.

We then performed *in vivo* tumor formation assays to evaluate the effect of the G3842A mutation on tumor formation and proliferation (Figure 8(a)). The results showed no difference in tumor weight (Figure 8(b)) and volume (Figure 8(c)) between groups of nude mice injected with WT cells and WT + NAC cells, whereas tumor weight and volume were drastically decreased in mice injected with MUT + NAC cells, relative to mice injected with only MUT cells (Figures 8(b) and 8(c)). These results indicated that the G3842A mutation promoted tumor growth *in vivo* in an ROS-dependent manner.

## 4. Discussion

Cytoplasmic hybrid technology is one of the major tools used in mtDNA research. The mtDNA-less p0 human osteosarcoma 143b cells are a widely accepted cellular model for studying the influence of mtDNA mutations on cellular function in various diseases, including Parkinson's disease [31], type 2 diabetes [32], and cancer metastasis [33]. Therefore, in the present study, we also employed the 143b cell line to study the link between the mtDNA mutation G3842A and thyroid cancer.

As shown in our study, mtDNA mutations that are disruptive to the mitochondrial structure and function could be involved in tumorigenicity. However, the pathways induced by mtDNA mutations that promote cancers vary, although disruptive mtDNA mutations all result in mitochondrial dysfunction. Here, we found that the G3842A mutation significantly decreased the OXPHOS function and promoted tumorigenicity, at least partially though ROS-mediated activation of ERK1/2 signaling. While Petros et al. [7] showed that the mtDNA mutation T8993G increases prostate tumor growth by elevating ROS levels and Singh et al. [34] found that mitochondrially encoded CO1 mutations may promote the cancer phenotype by modulating apoptotic processes and DNA-damage-response pathways. Therefore, it is necessary to investigate the pathogenic mechanism of each significant mitochondrial DNA mutation.

Mitochondrial OXPHOS function is necessary to support cancer growth, as mitochondria regulates the process of apoptosis [35] and autophagy [36], which provide an appropriate microenvironment for tumor cells. In the present study, colony formation assays showed increased clone numbers in G3842A mutant cells as compared with WT cells under both 1% and 20% O_2_ conditions, although growth curves showed a higher number of G3842A cells under only 1% O_2_ condition relative to WT cells. One possible explanation is that the G3842A mutation increases cell proliferation, as well as apoptosis and autophagy, under 20% O_2_ condition. As the cell growth curve is affected by both cell proliferation and apoptosis, complex I is the main site of ROS production in the mitochondria and ROS is an activator of autophagy and apoptosis signaling. Under 20% O_2_ condition, impaired complex I activity in G3842A-mutated cells generated higher ROS levels to activate autophagy and apoptosis, whereas under 1% O_2_ condition, oxygen flow and respiration were limited, and the ROS levels were insufficient to activate autophagy and apoptosis. Another possibility is that under 20% O_2_ condition, the G3842A mutation promotes tumorigenicity in a proliferation-independent manner. In addition to rapid proliferation, cancer cells are also characterized by immortality, increased mobility, and loss of contact inhibition. In the present study, the G3842A mutation accelerated clonal formation, indicating that this mutation may exert a positive effect on hypersensitivity to contact inhibition. Additionally, cell migration and invasion assays showed that G3842A significantly promote metastasis. These alterations may account for the tumorigenic potential of the G3842A mutation.

Mitochondria play an important role in cancer initiation and development [37], being also associated with the degree of decline in mitochondrial function. In the present study, the mutation frequency of G3842A was 54%, whereas that of G3842A identified in previous studies on FTC was 38% [15] and on hepatocellular carcinomas was 88.9% [19]. Mitochondrial function varies in cancer with heteroplasmy [38], genetics, environmental factors, and the tissue of origin [39]. Notably, even the same mtDNA mutation can produce different results.

Few studies have evaluated the relationship between mtDNA mutations and the tumorigenicity of thyroid cancer. Our findings showed for the first time that the mtDNA mutation G3842A promotes tumorigenicity. Additionally, we elucidated the associated mechanisms, which revealed that the G3842A mutation impairs mitochondrial function and increases ROS generation, which activates the ERK1/2 signaling pathway to facilitate cancer cell proliferation, invasion, and migration. Analyses of sequencing and experimental results indicated that disruptive mtDNA mutations in thyroid can be high-risk factors for promoting tumorigenicity. However, this work has certain limitations. We did not determine whether altered levels of Ca^2+^ and the NAD/NADH ratio also regulate ERK1/2 signaling and the tumorigenic potential of G3842A mutant cells. Moreover, although P38 and JNK pathways were also altered by the G3842A mutation (though to a lesser degree than that of ERK1/2), we did not examine the effects of P38 and JNK on tumorigenicity in these cells. Additionally, we only used a general ROS scavenger (NAC) to provide indirect evidence that excessive ROS generation was necessary for tumor development. Therefore, it remains unclear whether elevated ROS act as signaling molecules or a trigger for the oxidative stress. Both activities can contribute to tumor development, where a small increase in ROS levels is sufficient to activate signaling pathways that initiate biological processes to promote cancer development (e.g., the ERK1/2 pathway in this study), whereas high ROS levels can also damage DNA, proteins, and/or lipids, leading to a series of genetic and metabolic changes that contribute to carcinogenesis [40]. Therefore, further tests involving markers of oxidative stress and levels of antioxidant enzymes are needed to define a situation of oxidative stress in this context [41]. Furthermore, if the G3842A mutation causes oxidative stress, it will need to be demonstrated whether and how this process is involved in tumor development. Finally, further studies of the G3842A mutation are needed to elucidate its precise role in thyroid carcinoma.

## Data Availability

The data used to support the findings of this study are available from the corresponding author upon request.

## Supplementary Materials

Supplementary Materials Supplementary Table S1: analysis of mtDNA mutations identified in patients with thyroid carcinomas. Supplementary Table S2: clinical characteristics of patients with thyroid carcinomas.
