# Supplementary material for: Thyroid Cancer-Associated Mitochondrial DNA Mutation G3842A Promotes Tumorigenicity via ROS-Mediated ERK1/2 Activation
Source: Oxid Med Cell Longev. 2022 Mar 15;2022:9982449. doi: 10.1155/2022/9982449 (PMC9020963; doi:10.1155/2022/9982449)
Supplement: Supplementary Materials — Supplementary Table S1: analysis of mtDNA mutations identified in patients with thyroid carcinomas. Supplementary Table S2: clinical characteristics of patients with thyroid carcinomas. [file 9982449.f1.docx]

**S1:Analysis of mtDNA mutations identified in patients with thyroid carcinomas.**

| Patient | Gene | Base change | Blood | Adjacent tissue | Carcinoma  tissue | MUT  Type | Aminoacid change | Polarity of Aminoacid | Evolutionary conservation | Novelty |
| --- | --- | --- | --- | --- | --- | --- | --- | --- | --- | --- |
| Tc253 | ND1 | 3571insC | T | T | C | F | L89insC | - | * | Y |
| Tc254 | ND1 | G3842A | G | G | A | N | W179Ter | - | * | Y |
| Tc286 | ND1 | C3416A | C | C | A | M | P37H | Non→Alk | * | N |
| Tc296 | ND1 | G3380A | G | G | A | M | R25Q | Alk→Non | * | N |
| Tc325 | ND2 | G4818A | G | G | G/A | M | E117K | Acid→Alk | * | Y |
| Tc303 | ND2 | G4720A | G | G | A | N | W84Ter | - | * | N |
| Tc289 | ND3 | T10252C | T | T | C | M | F65S | Non→Neu | * | N |
| Tc261 | ND4 | T11046C | T | T | C | M | L96P | Non→Non | * | Y |
| Tc351 | ND4 | G11169A | G | G | G/A | M | G137D | Non→Alk | * | Y |
| Tc251 | ND4 | G11423A | G | G | A | M | E222K | Acid→Alk | * | N |
| Tc296 | ND4 | T11441C | T | T/C | T/C | M | S228P | Non→Neu | * | Y |
| Tc335 | ND4 | 10953insC | T | T | C | F | L65insC | - | * | N |
| Tc259 | ND4 | 11038DelA | A | A | C | F | K93delA | - | * | N |
| Tc266 | ND4 | T11613C | T | T | C | M | L285P | Alk→Non | * | N |
| Tc261 | ND4L | G10653A | G | G/A | A | M | A62T | Non→Neu | - | N |
| Tc296 | ND5 | T12467C | T | T | T/C | M | F44C | Non→Neu | * | N |
| Tc356 | ND5 | T13616A | T | T | A | M | I427N | Non→Acid | - | N |
| Tc256 | ND5 | T13706A | T | T | A | M | L457P | Non→Non | * | N |
| Tc281 | ND5 | C13250T | C | C | T | M | S305F | Neu→Non | * | N |
| Tc336 | ND5 | C13784A | C | C | A | M | P483H | Non→Alk | * | N |
| Tc346 | ND5 | 12382DelA | A | A | T | F | I16delA | - | - | N |
| Tc322 | ND5 | 12384insT | C | C | T | F | I16insT | - | - | N |
| Tc337 | ND6 | G14453A | G | G | G/A | M | A74T | Non→Neu | * | N |
| Tc341 | CytB | G14888A | G | G | A | N | G48Ter | - | * | N |
| Tc331 | CytB | G14973A | G | G | A | M | G76D | Non→Acid | * | Y |
| Tc315 | CytB | G15135A | G | G | A | M | G130D | Non→Acid | * | N |
| Tc262 | CytB | T15144C | T | T | C | M | L133P | Non→Non | * | N |
| Tc277 | CytB | T10159C | T | T | C | M | I115T | Non→Neu | * | N |
| Tc276 | COX1 | G6321A | G | G/A | A | N | G140Ter | - | * | N |
| Tc330 | COX1 | G6708A | G | G | A | N | G269Ter | - | * | N |
| Tc269 | COX1 | G6727A | G | G | A | N | W275Ter | - | * | N |
| Tc342 | COX1 | G6789A | G | G | A | N | G296Ter | - | * | N |
| Tc304 | COX1 | G6729A | G | G | A | M | A276T | Non→Neu | * | Y |
| Tc252 | COXI | T6229C | T | T | C | M | L109P | Non→Non | * | Y |
| Tc356 | COX1 | G6958A | G | G | A | M | G352D | Non→Acid | * | N |
| Tc342 | COX1 | G7075A | G | G | A | M | G391E | Non→Acid | * | Y |
| Tc301 | COX2 | A7718G | A | A/G | A/G | M | T53A | Neu→Non | * | Y |
| Tc304 | ATP6 | T9128C | T | T/C | T/C | M | I 201T | Non→Neu | * | Y |

Tc, thyroid carcinoma; Non, nonpolar hydrophobicity; Neu, neutral polar; Acid, acid polar; Alk, alkaline polar; M, missense mutation; N, nonsense mutation; F, frameshift mutation. Evolutionary conservation analysis: the amino acid sequences were compared with the published sequences of human, chimpanzee, rat, mice, dog, cow, chicken, zebra fish, xenopus, and fruit fly. *, high conservatism of evolution; -, not a conserved site in evolution.

**S2: Clinical characteristics of patients with thyroid carcinomas.**

| **Patient** | **Gender** | **Age** | **Family**  **history** | **Lymphatic**  **metastasis** | **Tumor size**  **（cm x cm）** | **FT4**  **(pmol/L)** | **TSH**  **(mlU/L)** | **FT3**  **(pmol/L)** | **Pathological diagnosis** |
| --- | --- | --- | --- | --- | --- | --- | --- | --- | --- |
| Tc251 | Female | 41 | - | Y | 1.0x1.0 | 15.8 | 0.34 | 3.45 | papillary thyroid carcinoma |
| Tc252 | Male | 41 | - | N | 1.0x1.0 | 11.7 | 1.75 | 3.84 | papillary thyroid carcinoma |
| Tc253 | Female | 54 | - | Y | 1.0x1.0 | 15.4 | 0.68 | 2.98 | papillary thyroid carcinoma |
| Tc254 | Female | 52 | - | Y | 1.0x1.0 | 3.11 | 1.04 | 2.68 | papillary thyroid carcinoma |
| Tc256 | Male | 58 | - | N | 1.5x1.0 | 12.4 | 1.14 | 4.54 | papillary thyroid carcinoma |
| Tc257 | Female | 46 | - | N | 0.8x0.8 | 13.9 | 1.63 | 4.09 | papillary thyroid carcinoma |
| Tc258 | Female | 46 | - | Y | 1.0x1.0 | 12.8 | 1.79 | 3.68 | papillary thyroid carcinoma |
| Tc259 | Female | 57 | - | N | 09x0.9 | 15.22 | 1.53 | 4.48 | papillary thyroid carcinoma |
| Tc261 | Female | 43 | - | Y | 1.5x1.2 | 15.16 . | 1.67 | 3.75 | papillary thyroid carcinoma |
| Tc262 | Female | 56 | - | N | 0.4x0.4 | 193 | 0.0002 | 6.04 | papillary thyroid carcinoma |
| Tc264 | Female | 50 | - | N | 12x1.0 | 15.86 . | 1.33 | 4.53 | papillary thyroid carcinoma |
| Tc266 | Female | 49 | - | N | 0.8x0.8 | 6.12 | 0.93 | 4.33 | papillary thyroid carcinoma |
| Tc269 | Female | 51 | - | N | 0.35x0.3 | 4.45 | 0.8 | 4.72 | papillary thyroid carcinoma |
| Tc270 | Female | 54 | - | Y | 12x1.2 | 18.5 | 0.13 | 4.22 | papillary thyroid carcinoma |
| Tc272 | Female | 72 | - | Y | 1.0x1.0 | 13.2 | 1.03 | 2.65 | papillary thyroid carcinoma |
| Tc274 | Male | 35 | - | Y | 0.5x0.5 | 11.1 | 1.48 | 5.94 | papillary thyroid carcinoma |
| Tc276 | Female | 37 | - | N | 0.5x0.5 | - | - | - | papillary thyroid carcinoma |
| Tc277 | Male | 37 | - | N | 1.0x1.0 | 13.8 | 0.73 | 4.54 | papillary thyroid carcinoma |
| Tc281 | Female | 56 | - | Y | 1.2x1.2 | 18.16 | 3.48 | 4.85 | papillary thyroid carcinoma |
| Tc286 | Female | 41 | - | Y | 0.8x0.8 | 13.57 | 0.92 | 4.29 | papillary thyroid carcinoma |
| Tc289 | Female | 39 | - | Y | 1.0x0.8 | 11.3 | 1.44 | 3.87 | papillary thyroid carcinoma |
| Tc295 | Female | 44 | - | N | 0.5x0.3 | 17.11 | 1.55 | 3.9 | papillary thyroid carcinoma |
| Tc296 | Female | 47 | - | Y | 2.0x2.0 | 18.1 | 1.1 | 4.73 | papillary thyroid carcinoma |
| Tc297 | Female | 81 | - | Y | 1.0x1.0 | 17.65 | 0.97 | 3.05 | papillary thyroid carcinoma |
| Tc298 | Female | 46 | - | Y | 0.6x0.6 | 16.2 | 1.44 | 4.21 | papillary thyroid carcinoma |
| Tc301 | Male | 14 | - | Y | 2.0x2.0 | 14.4 | 1.35 | 5.21 | papillary thyroid carcinoma |
| Tc302 | Female | 44 | - | N | 1.0x1.0 | 14.4 | 1.64 | 5.21 | papillary thyroid carcinoma |
| Tc303 | Female | 54 | - | Y | 1.0x0.6 | 15.72 | 2.59 | 3.97 | papillary thyroid carcinoma |
| Tc304 | Male | 47 | - | Y | 0.5x0.5 | 14.6 | 2.95 | 3.62 | papillary thyroid carcinoma |
| Tc306 | Female | 36 | - | N | 1.0x1.0 | 13.29 | 1.17 | 3.44 | follicular thyroid carcinoma with capsular invasion |
| Tc310 | Female | 26 | - | N | 1.0x1.0 | 13.94 | 2.8 | 3.89 | papillary thyroid carcinoma |
| Tc311 | Female | 50 | - | N | 0.5x0.3 | 15.52 | 0.34 | 3.39 | papillary thyroid carcinoma |
| Tc315 | Male | 63 | - | N | 0.3x0.3 | 11.7 | 0.58 | 4.56 | papillary thyroid carcinoma |
| Tc316 | Female | 36 | - | Y | 1.0x1.0 | 15.54 | 1.9 | 4.23 | papillary thyroid carcinoma |
| Tc322 | Female | 42 | - | N | 1.2x1.0 | 14.44 | 2.1 | 3.66 | papillary thyroid carcinoma |
| Tc324 | Female | 55 | Daughter | N | 0.3x0.3 | 14.9 | 2.11 | 4.19 | papillary thyroid carcinoma |
| Tc325 | Female | 60 | - | Y | 0.6x0.6 | 17.88 | 0.05 | 5.34 | papillary thyroid carcinoma |
| Tc329 | Male | 34 | - | N | 2.5x2.5 | 18.2 | 0.89 | 3.97 | papillary thyroid carcinoma |
| Tc330 | Female | 42 | - | Y | 0.5x0.5 | 15.66 | 1.76 | 4.5 | papillary thyroid carcinoma |
| Tc331 | Male | 48 | - | N | 0.5x0.5 | 12.9 | 1.08 | 4.97 | papillary thyroid carcinoma |
| Tc333 | Female | 61 | - | Y | 0.5x0.5 | 16.9 | 1.59 | 3.69 | papillary thyroid carcinoma |
| Tc335 | Male | 35 | - | N | 0.5x0.5 | 15.3 | 0.51 | 4.48 | papillary thyroid carcinoma |
| Tc336 | Female | 43 | - | N | 0.3x0.3 | 19.78 | 3.61 | 5.59 | papillary thyroid carcinoma |
| Tc337 | Female | 62 | - | N | 0.5x0.5 | 14.8. | 0.24 | 4.3 | papillary thyroid carcinoma |
| Tc338 | Female | 58 | - | N | 1.5x1.5 | 13.19 | 0.84 | 3.94 | papillary thyroid carcinoma |
| Tc339 | Female | 34 | - | N | 0.5x0.5 | 13.2 | 1.3 | 3.43 | papillary thyroid carcinoma |
| Tc341 | Female | 62 | - | N | 0.8x0.8 | 15.8 | 0.66 | 4.75 | papillary thyroid carcinoma |
| Tc342 | Male | 36 | - | N | 1.2x1.2 | 17.19 | 0.6 | 4.21 | papillary thyroid carcinoma |
| Tc344 | Female | 49 | - | N | 1.0x1.0 | 13.5 | 1.47 | 5.21 | papillary thyroid carcinoma |
| Tc346 | Female | 51 | - | N | 1.0x0.8 | 15.5 | 0.32 | 4.46 | papillary thyroid carcinoma |
| Tc347 | Female | 55 | - | N | 1.2x1.0 | 10.2 | 5.43 | 6.66 | papillary thyroid carcinoma |
| Tc349 | Female | 40 | - | N | 0.5x0.5 | 14.4 | 0.97 | 4.51 | papillary thyroid carcinoma |
| Tc351 | Female | 60 | - | N | 1.2x1.0 | 14.2 | 5.03 | 4.75 | papillary thyroid carcinoma |
| Tc355 | Female | 68 | - | Y | 1.0x1.0 | 21.53 | 0.05 | 5 | papillary thyroid carcinoma |
| Tc356 | Male | 47 | - | N | 0.4x0.4 | 15.1 | 3.39 | 4.15 | papillary thyroid carcinoma |
| Tc267 | Female | 52 | - | Y | 0.8x0.8 | - | - | - | papillary thyroid carcinoma |
| Tc282 | Male | 69 | - | Y | 1.2x1.2 | 12.87 | 1.96 | 4.48 | papillary thyroid carcinoma |
| Tc283 | Female | 53 | - | Y | 1.0x1.0 | 13 | 4.67 | 4.23 | papillary thyroid carcinoma |
| Tc334 | Female | 46 | - | N | 0.3x0.3 | 16.5 | 0.97 | 3.98 | papillary thyroid carcinoma |
| Tc353 | Female | 45 | - | Y | 0.8x0.8 | - | - | - | papillary thyroid carcinoma |
